# Identification of novel and conserved microRNAs in *Panax notoginseng* roots by high-throughput sequencing

**DOI:** 10.1186/s12864-015-2010-6

**Published:** 2015-10-22

**Authors:** Rongchang Wei, Deyou Qiu, Iain W. Wilson, Huan Zhao, Shanfa Lu, Jianhua Miao, Shixin Feng, Longhua Bai, Qinghua Wu, Dongping Tu, Xiaojun Ma, Qi Tang

**Affiliations:** Institute of Medicinal Plant Development, Chinese Academy of Medical Sciences & Peking Union Medical College, Beijing, 100193 China; Guangxi Botanical Garden of Medicinal Plants, Nanning, 530023 China; Hunan Provincial Key Laboratory of Crop Germplasm innovation and Utilization and National Chinese Medicinal Herbs (Hunan) Technology Center, Hunan Agricultural University, Changsha, 410128 China; Department of Molecular Biology, The Research Institute of Forestry, Chinese Academy of Forestry, Beijing, 100091 China; CSIRO Agriculture, PO Box 1600, Canberra, ACT 2001 Australia

**Keywords:** *Panax notoginseng*, MicroRNA, High-throughput sequencing, qRT-PCR, Saponins

## Abstract

**Background:**

MicroRNAs (miRNAs) are small, non-coding RNAs that are important regulators of gene expression, and play major roles in plant development and their response to the environment. Root extracts from *Panax notoginseng* contain triterpene saponins as their principal bioactive constituent, and demonstrate medicinal properties. To investigate the novel and conserved miRNAs in *P. notoginseng*, three small RNA libraries constructed from 1-, 2-, and 3-year-old roots in which root saponin levels vary underwent high-throughput sequencing.

**Methods:**

*P. notoginseng* roots, purified from 1-, 2-, and 3-year-old roots, were extracted for RNA, respectively. Three small libraries were constructed and subjected to next generation sequencing.

**Results:**

Sequencing of the three libraries generated 67,217,124 clean reads from *P. notoginseng* roots. A total of 316 conserved miRNAs (belonging to 67 miRNA families and one unclassified family) and 52 novel miRNAs were identified. MIR156 and MIR166 were the largest miRNA families, while miR156i and miR156g showed the highest abundance of miRNA species. Potential miRNA target genes were predicted and annotated using Cluster of Orthologous Groups, Gene Ontology, and Kyoto Encyclopedia of Genes and Genomes. Comparing these miRNAs between root samples revealed 33 that were differentially expressed between 2- and 1-year-old roots (8 increased, 25 decreased), 27 differentially expressed between 3- and 1-year-old roots (7 increased, 20 decreased), and 29 differentially expressed between 3- and 2-year-old roots (8 increased, 21 decreased). Two significantly differentially expressed miRNAs and four miRNAs predicted to target genes involved in the terpenoid backbone biosynthesis pathway were selected and validated by quantitative reverse transcription PCR. Furthermore, the expression patterns of these six miRNAs were analyzed in *P. notoginseng* roots, stems, and leaves at different developmental stages.

**Conclusions:**

This study identified a large number of *P. notoginseng* miRNAs and their target genes, functional annotations, and gene expression patterns. It provides the first known miRNA profiles of the *P. notoginseng* root development cycle.

**Electronic supplementary material:**

The online version of this article (doi:10.1186/s12864-015-2010-6) contains supplementary material, which is available to authorized users.

## Background

The root of *Panax notoginseng* (Burk.) F. H. Chen, also known as Radix Notoginseng or Sanchi, is a well-known traditional Chinese medicine belonging to the genus *Panax* and family Araliaceae. *P. notoginseng* has been cultivated and used medicinally since ancient times for its remarkable and valuable hemostatic effects [1]. This species is not normally found in the wild, but is cultivated commercially in Wenshan County (Yunnan Province), and Jingxi County (Guangxi Province), China [2]. Clinical trials indicate that *P. notoginseng* root extracts possess anti-hypertensive, anti-oxidant, anti-thrombotic, anti-atherosclerotic, hepatoprotective, and neuroprotective properties [3–5].

The principal bioactive constituents of the herb are triterpene saponins, which are dammarane-type glycosides [3–5]. More than 60 variant dammarane-type saponins have been isolated and identified from *P. notoginseng* including notoginsenosides, ginsenosides, and gypenosides [6]. However, the oleanane-type saponin ginsenoside Ro has not been isolated from this species, although it exists in other major *Panax* plants such as *P. ginseng* and *P. quinquefolius* [7]. The *P. notoginseng* saponins (PNS) mainly contain ginsenoside Rg_1_, ginsenoside Rb_1_, and notoginsenoside R_1_ according to the Chinese Pharmacopoeia (2010 Edition). PNS content and composition differs between tissues and plant age, with the highest PNS levels found in roots [6].

Although the pharmacological importance of PNS is well established, the biosynthetic enzymes involved in their synthesis and their regulation is not fully understood. As steroids involved in plant defense they are thought to derive from pathways that lead to the synthesis of isoprenoids. These precursors of PNS are synthesized by the mevalonic acid (MVA) pathway in the cytosol and the methylerythritol phosphate (MEP) pathway in the plastid, resulting in the synthesis of 2,3-oxidosqualene [8]. Subsequent modification of 2,3- by multiple oxidations (e.g. mediated by cytochrome P450-dependent monooxygenases) and glycosylation results in a wide variety of PNSs. Although most of MVA-pathway genes in *P. notoginseng* have not been functionally characterized, their corresponding sequence homologues have been identified. Recently a large-scale transcriptome analysis of *P. notoginseng* leaves, roots and flowers identified 270 unigenes putatively involved in triterpene saponin biosynthesis and a large number of cytochrome P450 and glycosyltransferease-like genes that may be involved conversion of triterpene saponin backbone into different PNSs [9].

microRNAs (miRNAs), which are approximately 19–24 nucleotides in length, are a class of small endogenous non-coding RNAs (sRNA) that originate from primary transcripts of stem–loop structures [7]. In plants, miRNAs regulate target gene expression either through direct endonucleolytic cleavage or translational inhibition at the post-transcriptional level [10]; under certain circumstances, they lead to methylation at the transcriptional level [11–13]. Increasing evidence indicates that miRNAs play important regulatory roles in diverse biological and metabolic processes such as plant development, abiotic stress responses, and pathogen defense [14].

Plant miRNAs have been studied extensively using computational and experimental methods since the discovery of *Arabidopsis* miRNAs in 2002 [15–18]. To date, a total of 25,141 mature miRNA sequences from 193 different species (ranging from viruses to humans) have been identified according to the miRBase database (release 19.0, August 2012). However, there is currently no available information about *P. notoginseng* miRNAs. In this study, three sRNA libraries from *P. notoginseng* roots of different ages were constructed and analyzed by high-throughput sequencing (Illumina Genome Analyzer) [19–22]. We identified a total of 316 conserved miRNAs and 52 novel miRNAs from the three sRNA datasets. To investigate the functions of the novel miRNAs, the Cluster of Orthologous Groups (COG) annotation, Gene Ontology (GO) enrichment analysis, and Kyoto Encyclopedia of Genes and Genomes (KEGG) pathway mapping of predicted targets were performed [23]. Finally, the expression of six miRNAs was validated in the root samples and their expression patterns at different developmental stages and in different tissues of *P. notoginseng* were determined using quantitative reverse transcription (RT)-PCR.

## Results

### Triterpene saponin content analysis

High-performance liquid chromatography (HPLC) was performed on 1-, 2- and 3-year-old *P. notoginseng* root, stem, and leaf samples to quantify saponin levels. As expected, the levels of *P. notoginseng* saponins ginsenoside Rg_1_, ginsenoside Rb_1_, and notoginsenoside R_1_ were highest in the roots, with a large increase observed in saponin content between 1-year-old (1y) and 2-year-old (2y) roots. The highest saponin levels were found in 3-year-old roots (3y) (Additional file 1: Table S1). Stem PNS levels displayed an inverse concentration profile to roots, with decreasing levels of saponin seen with increasing age. Leaf samples possessed the lowest PNS levels compared with root and stem samples, with the highest PNS levels found in 2y samples. Pairwise analyses identified significant differences between triterpene saponin levels of root samples in different years (*P* ≤ 0.01), with the exception of R_1_ levels between 2y and 1y roots.

### Features of *P. notoginseng* sRNA populations

**Fig. 1:**
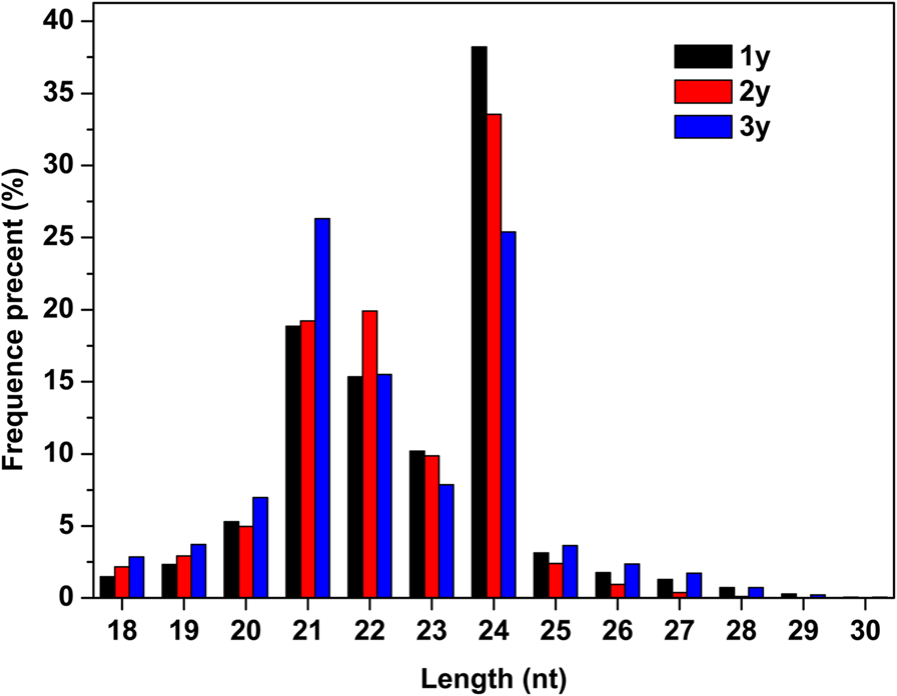
Length distribution and abundance of sRNAs in *Panax notoginseng*

To examine *P. notoginseng* root miRNAs, three sRNA libraries were generated from 1y, 2y, and 3y old root (herb) samples. The libraries were sequenced by Illumina sRNA deep sequencing technology. Sequences of the 3 libraries were combined and then analyzed the features of miRNAs and identified novel and conserved miRNAs. In total, 69,361,627 raw root sequence reads were obtained. After removing contaminants, low quality sequences, and those reads shorter than 18 nt, 67,217,124 (97.36 %) clean reads of 18–30 nt were used for further analysis (Table 1). Among the selected reads, 12,754,484 sequences from *P. notoginseng* roots mapped perfectly to the transcriptome, amounting to 18.98 %, of the total reads (Table 2). Additionally, 6,129,134 reads in *P. notoginseng* roots were found to be highly similar to previously identified miRNAs from other species. The remaining sequences consisted of other RNA types, including non-coding RNA, transfer (t)RNA, ribosomal (r)RNA, small nuclear (sn)RNA, small nucleolar (sno)RNA, and novel miRNAs (Table 2). The size distributions of sRNAs were uneven between the three root libraries, but the majority ranged in length from 20–24 nt (Fig. 1).

**Table 1:** Statistics of high-through sequencing results of *Panax notoginseng* small RNAs

	Read number	Read percentage (%)
total reads	69361627	
high quality	69041414	100
adapter 3 null	12057	0.02
insert null	11535	0.02
adapter 5 contaminants	136781	0.2
smaller than 18 nt	1658945	2.4
polyA	4972	0.01
clean reads	67217124	97.36

**Table 2:** Summary of Illumina small RNA deep sequencing data for small RNAs of *Panax notoginseng*

Category	Unique sRNAs	%	Total sRNAs	%
total	15267563	100	67217124	100
mapping to transcriptome	1082863	7.09	12754484	18.98
miRNA	2028	0.01	6129134	9.12
rRNA	257687	1.69	8356595	12.43
tRNA	64444	0.42	2986789	4.44
snRNA	4853	0.03	10441	0.02
snoRNA	2484	0.02	6226	0.01
un-annotated	14936067	97.83	49727939	73.98

### Identification of conserved *P. notoginseng* root miRNAs

To investigate the expression of conserved miRNAs in *P. notoginseng* root, sRNA sequences from the three root libraries were compared with known mature miRNAs and precursors in miRBase 19.0. A total of 316 conserved miRNAs (belonging to 67 miRNA families and one unclassified family) were identified in *P. notoginseng* from these three sRNA datasets (Additional file 2: Table S2). The frequency of these miRNAs varied widely between families. In most miRNA families, such as MIR1033, MIR1120, and MIR1510, only one conserved member was identified. However, some miRNA families possessed several members. For example, MIR156 was the largest family identified, with twenty four members, whereas twenty members were found in the MIR166 family, followed by MIR482, in which sixteen members were identified. The frequency of these miRNAs varied significantly between different miRNA families, with some having fewer than 100 sequence reads, while others possessed hundreds of thousands of reads.

The proportion of different categories of sRNAs often reflects the roles they play in a particular tissue or at different developmental stages, and their associated biological mechanisms [24–26]. The following 13 miRNAs were highly abundant in the combined library: miR156n, miR156j, miR156g, miR156q, miR156b, miR156, miR156i, miR166s, miR166l-3p, miR166k, miR166m, miR166u and miR166e. Each of these 13 miRNAs possessed more than 200,000 reads, whereas the majority of other miRNAs sequenced had approximately 500 reads. According to the sequence data, miR156i (2,154,952) and miR156g (2,154,952) were the most abundant miRNAs in *P. notoginseng* roots.

### Identification of novel miRNAs in *P. notoginseng* root

To identify novel miRNAs that may be specific to *P. notoginseng*, all unannotated sRNAs were searched against the publically available 96,704 unigene *P. notoginseng* transcriptome using MIREAP software. After searching for potential precursors (pre-miRNAs) and predicting their stem–loop hairpin secondary structures, a total of 52 novel miRNAs were identified in the three libraries (Additional file 3: Table S3). The novel miRNA sequences ranged in length from 20–23 nt, with a mode of 21 nt. The pre-miRNAs ranged in length from 72–363 nt, with an average of 122 nt. The average minimum folding free energy value of the hairpin structures was −39.71 kcal/mol in *P. notoginseng*, which is higher than that found in *Arabidopsis* (−59.50 kcal/mol) [27]. The structures of 52 new miRNA precursors are shown in Additional file 4: Figure S1. miRNA lengths and nucleotide preference distributions indicated that most miRNAs started with a 5′-U not 5′-G, which is consistent with typical miRNA sequence patterns [28].

### Target prediction and functional annotation

**Fig. 2:**
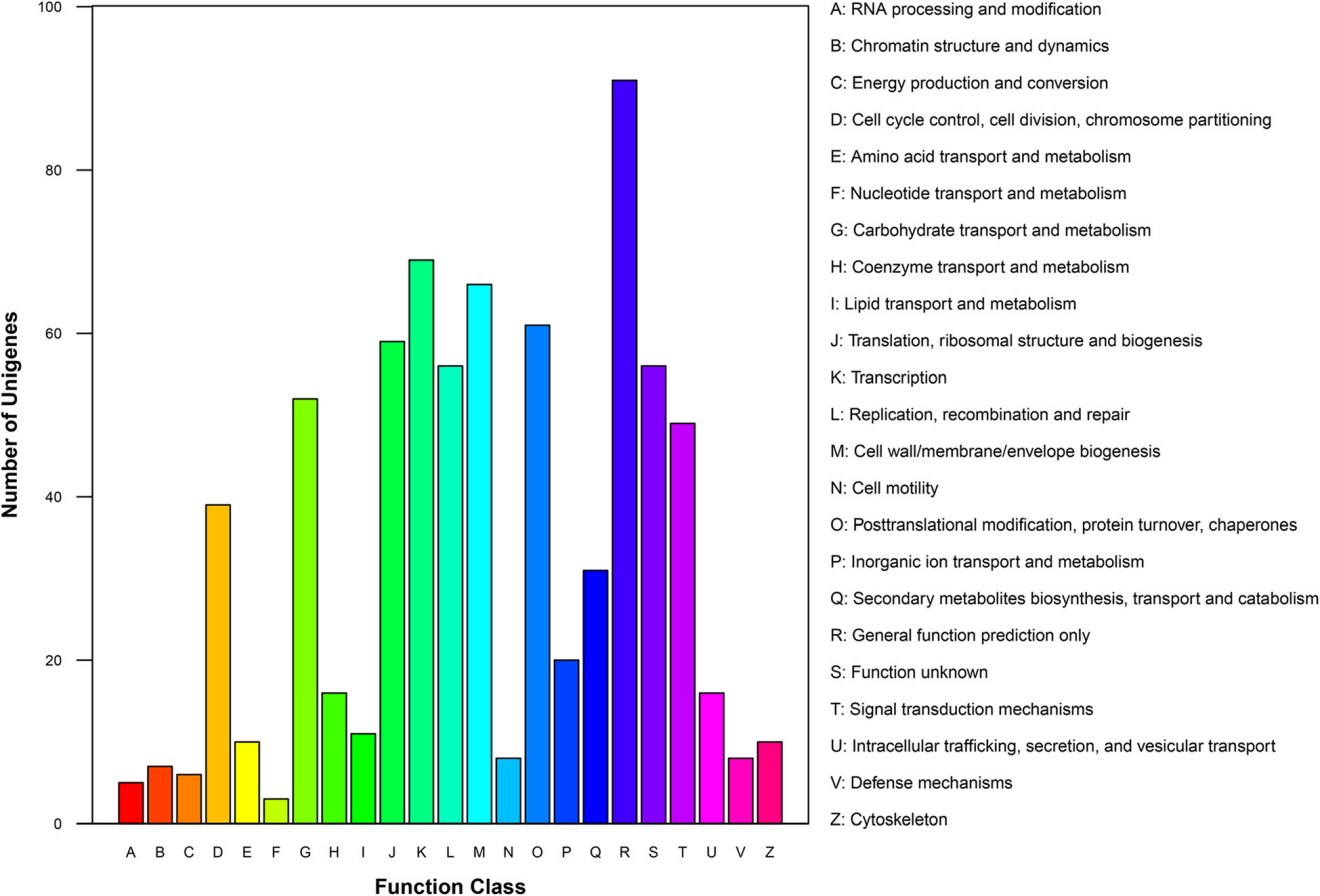
COG function classification of miRNA targets in *Panax notoginseng*

**Fig. 3:**
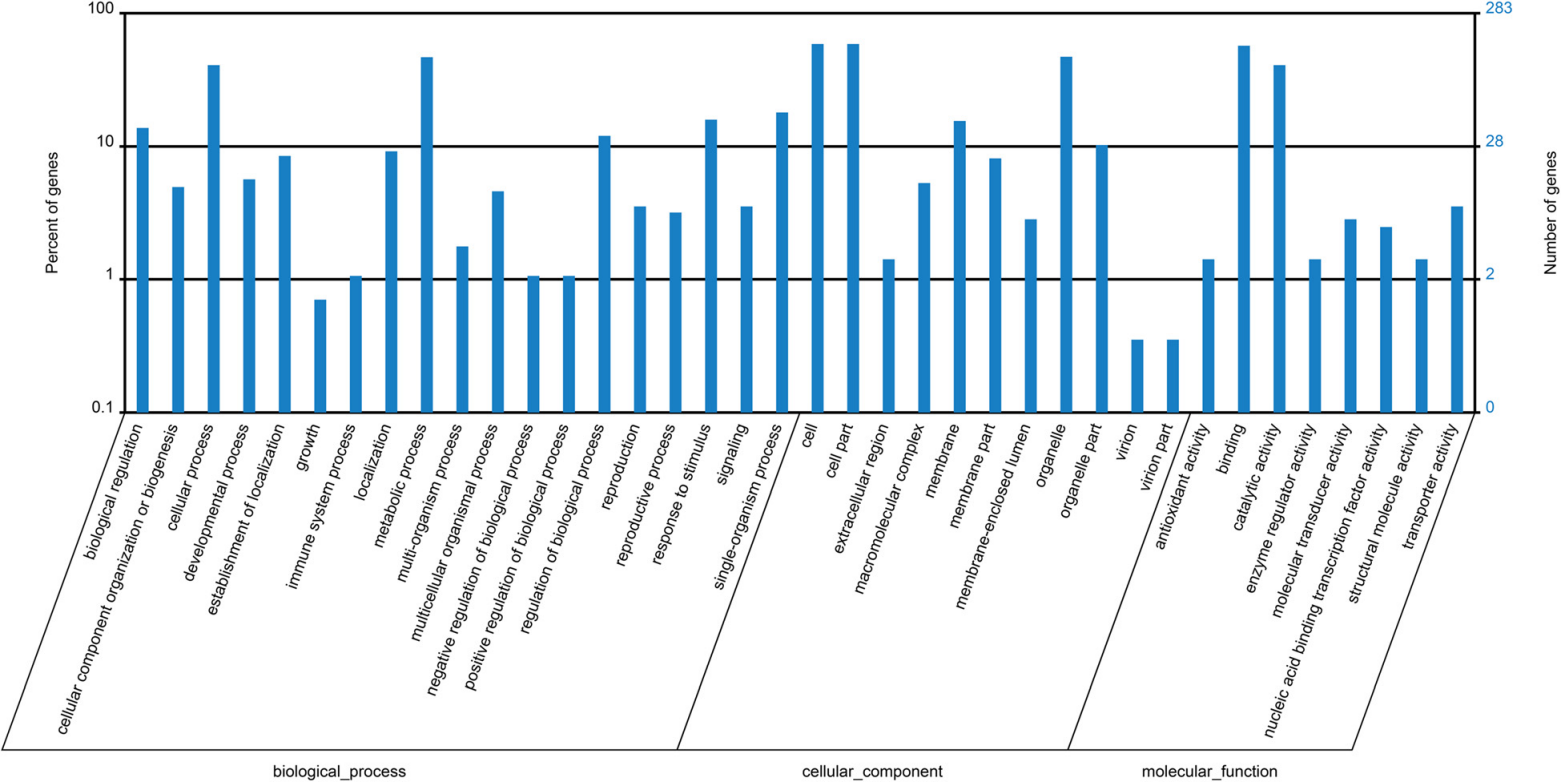
GO categories and distribution of miRNA targets in *Panax notoginseng*

We used psRNA Target software to predict putative target genes of novel and conserved miRNAs. A total of 803 putative target genes were identified in *P. notoginseng* roots (Additional file 5: Table S4). A total of 749 target gene sequences could be assigned to COG classifications, the database of orthologous gene products [29], and were classified into 23 groups. The largest of these groups was ‘general function prediction only’ followed by ‘transcription’ (Fig. 2). GO annotation enrichment showed that the most significantly enriched of biological_processes, cellular_components, and molecular_functions were metabolic process, cell/cell part, and binding, respectively (Fig. 3). Overall, 506 different pathways enriched with miRNA targets were found in *P. notoginseng*, and the most enriched included metabolic pathways (76 enzymes), spliceosome (61 enzymes) and biosynthesis of secondary metabolites (41 enzymes) (Additional file 6: Table S5).

Analysis of both novel and conserved miRNAs identified four miRNAs that were computationally predicted to target genes associated with the terpenoid backbone biosynthesis pathway. 3-hydroxy-3-methylglutaryl coenzyme A synthetase (*HMGS*), catalyses the reaction of acetoacetyl-coenzyme A to 3-hydroxy-3-methylglutaryl coenzyme A, the first committed enzyme of the mevalonate (MVA) pathway was predicted to be targeted by miR5021 and miR5293. miR5163 was predicted to target all-trans-nonaprenyl diphosphate synthase (geranyl–diphosphate specific), which may be involved with geranyl diphosphate synthase (*GPS*). *GPS* catalyses the condensation reaction of isopentenyl diphosphate (IPP) and dimethylallyl diphosphate (DMAPP), which product is geranyl diphosphate (GPP), the entry to the formation of terpene moiety. Finally, the putative target gene of novel_miR_27 was predicted to be 1-deoxy-D-xylulose-5-phosphate synthase (*DXS*), the first rate-limiting enzyme for sesquiterpene synthesis in the 2-C-Methyl-D-Erythritol-4-Phosphate (MEP) pathway in triterpene saponin biosynthesis. The synthesis of 1-deoxy-D-xylulose-5-phosphate (DXP) is catalyzed by the enzyme *DXS*.

### miRNAs differentially expressed in *P. notoginseng* root

Pairwise analysis of the miRNA abundance between root libraries identified 33 (8 increased, and 25 decreased), 27 (7 increased, and 20 decreased), and 29 (8 increased, and 21 decreased) significantly differentially expressed miRNAs (|log_2_ratio| ≥ 1, adjusted *P*-value ≤0.01) between 2y–1y, 3y–1y, and 3y–2y root samples, respectively (Additional file 7: Table S6). Interestingly, different expression levels were also observed among different members of the same miRNA family, namely those involved in tissue- or development stage-specific expression. For example, two members of the MIR156 family, miR156a and miR157a-3p, exhibited significantly differential expression between 2y–1y, although the fold-changes were lower at −3.7 and −2.5 log_2_ ratio, respectively.

### qRT-PCR validation and analysis of six miRNAs in *P. notoginseng*

**Fig. 4:**
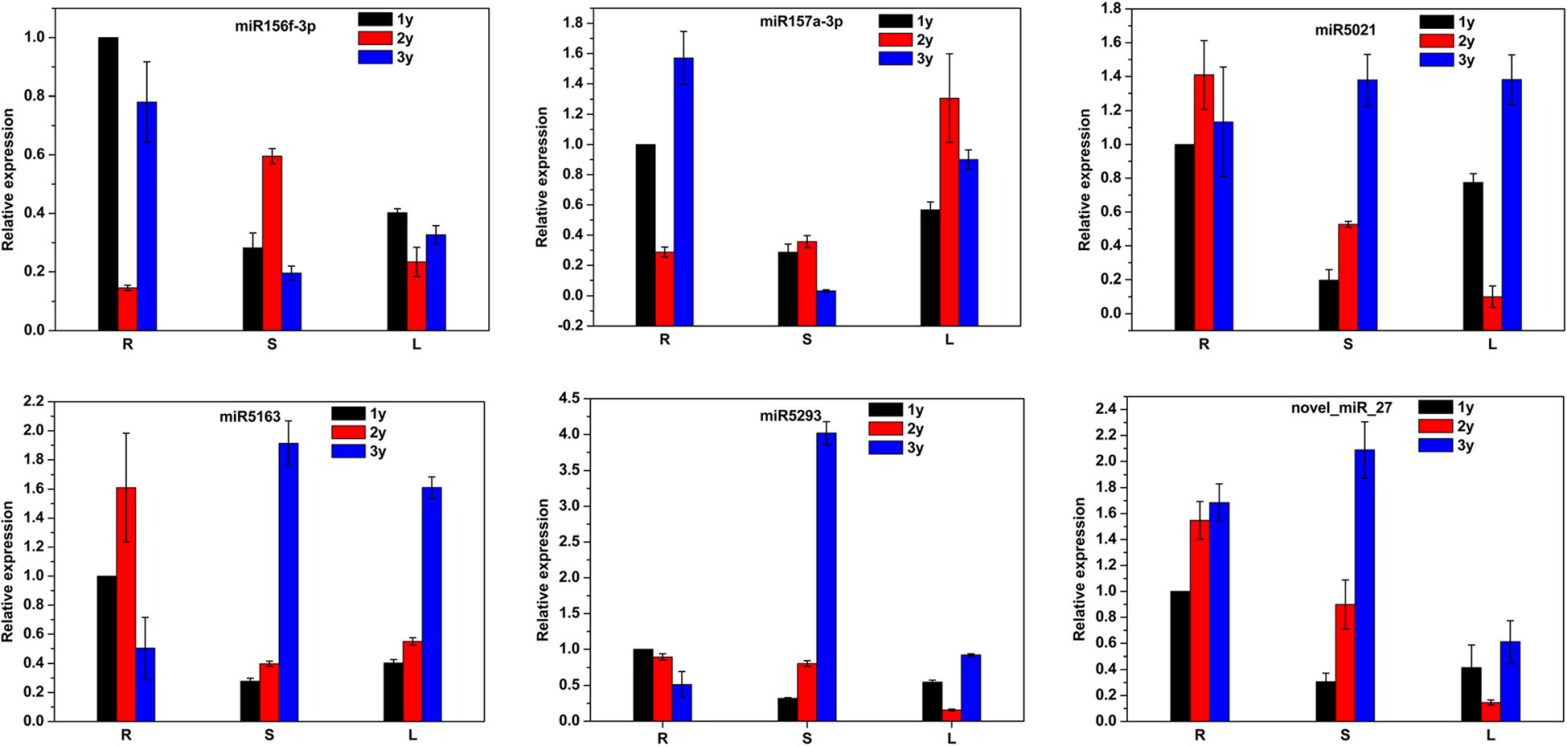
qRT-PCR validation of miRNAs in *Panax notoginseng*. Relative expression of miRNAs in roots (R), stems (S) and leaves (L) are shown. Reference gene was 18S rRNA. Normalized miRNA levels in 1y roots were arbitrarily set to 1

To validate the data obtained from high-throughput sequencing, two significantly differentially expressed miRNAs (miR156f-3p and miR157a-3p) and four miRNAs (miR5021, miR5163, miR5293, and novel_miR_27) predicted to target genes involved in the terpenoid backbone biosynthesis pathway were selected and their expression levels quantified using stem–loop qRT-PCR (Fig. 4). The results were consistent with deep sequencing data (Additional file 8: Figure S2). The majority of analyzed miRNAs showed age- and tissue-specific expression with predominant expression in the roots (Fig. 4). For example, miR5163 and miR5293 exhibited the lowest expression in roots of 3y plants, while miR157f-3p and novel_miR_27 showed high expression in 3y roots and relatively low expression in 1y and 2y roots. This indicates that developmental stage-specific regulation of miRNA expression occurs in *P. notoginseng*.

In the 1y sample, miR156f-3p, miR157a-3p, miR5021, miR5163, miR5293, and novel_miR_27 showed very similar expression patterns, with the highest levels expressed in roots, followed by leaves and stems. The expression patterns of miR5021, miR5293, and novel_miR_27 were also similar in the different tissues of 2y plants, with the highest expression observed in roots, followed by stems and leaves. miR156f-3p and miR157a-3p 3y expression patterns were consistent with that of the miR5163 2y sample, which showed the same patterns as the 1y sample. miR5021, miR5163, and miR5293 of the 3y sample exhibited similar expression patterns, with the lowest levels expressed in roots, followed by leaves and stems.

miR156f-3p and miR157a-3p are members of the same miRNA family. Their expression patterns were similar in the root and stem tissues of 1y and 3y plants. This implies that a functional relationship exists between miR156f-3p and miR157a-3p in these tissues at this developmental stage. However, their expression patterns differed from that observed in 2y tissues. miR156f-3p showed the highest expression in the stem, the lowest expression in the roots, and moderate expression in the leaves, whereas miR157a-3p exhibited the highest expression in the leaves, followed by the stem and roots. This suggests that miR156f-3p and miR157a-3p could be functionally different at 2y, although they belong to the same family.

## Discussion

High-throughput sequencing and bioinformatic approaches are efficient means for identifying miRNAs [24]. Many conserved plant miRNAs have been previously identified, although relatively few have been well characterized [22, 29, 30]. In this study, sRNA profiles from 1y, 2y, and 3y *P. notoginseng* roots were obtained, which represents the first major examination of miRNAs in this species. Sequence analysis showed that the majority of sRNAs ranged in length from 20–24 nt. These results are typical of Dicer-processed sRNA products and in keeping with the known 18–25 nt range for miRNAs [30, 31]. More than 60 million clean reads of 18–30 nt were obtained from each sRNA library. A total of 316 known miRNAs (belonging to 67 families and one unclassified family) and 52 novel miRNAs were identified in *P. notoginseng* roots. miR156a was one of frequently sequenced miRNAs. miR156a is also expressed abundantly in *Taxus chinensis* [27] and *Arachis hypogaea* [30].

Several miRNAs were computationally predicted to directly target known genes in the terpenoid backbone biosynthesis pathway, including *HMGS* and *DXS*. qRT-PCR analysis of these miRNAs in root, stem, and leaf material from 1y, 2y, and 3y tissues revealed complex expression profiles that showed good concordance with the sequencing data. miRNAs usually negatively regulate the expression of genes, therefore it would be expected that increased levels of a miRNA that targets biosynthetic genes in the PNS pathway may inversely correlate with PNS levels. For stem tissue, levels of miR5021, miR5163, miR5293, and novel_miR_27 all inversely correlated with total PNS content, whereas in leaf tissues miR5021, miR5293, and novel_miR_27 inversely correlated only with Rb_1_ content. However in the roots only miR5293 inversely correlated with PNS content as measured by HPLC. However, PNS biosynthesis is a complex process involving many triterpene saponins, so it is simplistic to discern simple correlations between miRNA expression and saponin content. Given the importance of triterpene saponins from *P. notoginseng,* further characterization of these miRNAs is required, because greater understanding of the regulatory mechanism of miRNAs in triterpene saponin biosynthesis could facilitate a miRNA-based biotechnology for manipulating PNS in both *in vitro* and *in vivo* systems.

Characterization of specific cytochrome P450s (CYP450s) involved in triterpene saponin biosynthesis in *P. notoginseng* will facilitate to elucidation of the triterpene saponin biosynthetic pathway. It is reported that some CYP450s participate in the oxidation of the dammarane skeleton at C-6 and the other at C-12 toward the production of protopanaxatriol and protopanaxadiol, respectively [32, 33]. Previous studies have characterized CYP716A12 from *Medicago truncatula* (CYP85 clan) [34], which was involved in triterpene saponin biosynthesis. Therefore, the novel_miR_47 in this paper, one of its putative targets was CYP716A12 (belonging to CYP85 clan), which might be a candidate enzyme involved in triterpene saponin biosynthesis in *P. notoginseng*.

Target gene prediction in this study showed that miRNAs can target transcription factors related to plant growth and development, including squamosa promoter-binding protein-like (SPL), APETALA2 (AP2), myeloblastosis related proteins (MYB), and WRKY proteins. Previous research indicated that miRNA families in different plants are highly conserved and perform analogous regulatory functions [35]. For example, miR156 was predicted to target the SPL transcription factor, which plays an important part in the developmental transition from the juvenile to adult phase of *Arabidopsis* and from the vegetative to reproductive phase during postembryonic development [36]. The AP2 transcription factor is involved in flowering time, while miR172 regulates *Arabidopsis* floral morphology [37], and targets AP2 in *P. ginseng* [38], *Brassica oleracea* [21], and the opium poppy [39]. In *Arabidopsis*, miR156 maintains the juvenile stage and prevents precocious flowering, whereas miR172 acts downstream of miR156 and promotes flowering [40], and miR172 targets and promotes the expression of *SPL* genes that are negative regulators of miR156 [37, 38, 41]. miR156 and miR172 exhibit contrasting development-specific expression patterns: the miR156 abundance declines during vegetative development, while that of miR172 correspondingly increases.

One of the larger groups of plant transcription factors is the MYB protein family. Members carry a conserved MYB DNA binding domain and play a regulatory role in developmental processes and defense responses [37, 41]. This study suggests that MYB proteins might be the target of miR5021 in *P. notoginseng*. Previous studies have also suggested that this occurs in *P. ginseng* [42] and *B. oleracea* [21]. Additionally, WRKY transcription factor family members are key players in responding to bacterial infections, wounding, and other stresses [39]. Interestingly, certain WRKY members are positive regulators of triterpene ginsenoside biosynthesis in *P. quinquefolius* [43]. Therefore, the conservation of putative target genes in *P. notoginseng* suggests that these transcription factors also share the same function in plant growth and development, including in triterpene ginsenoside biosynthesis, although this requires further experimental validation.

## Conclusions

The present study is the first report to examine the miRNA expression profiles of *P. notoginseng* roots. We identified a large number of conserved and novel miRNAs in *P. notoginseng*, as well as their target genes, functional annotations, and gene expression patterns. Four miRNAs were computationally predicted to directly target known genes in the terpenoid backbone biosynthesis pathway. Further investigation concerning the functions of these miRNAs should facilitate our understanding of the regulatory roles of miRNAs in regulating *P. notoginseng* triterpene saponin biosynthesis. Greater understanding of these regulatory mechanisms could facilitate miRNA-based biotechnology for manipulating saponin production in both *in vitro* and *in vivo* systems.

## Methods

### Plant material

*P. notoginseng* plants were grown in Jingxi County, Guangxi Province, China to 1y, 2y, and 3y. Leaves, stems, and roots were collected from all plants and immediately frozen in liquid nitrogen. The frozen samples were then stored at −80 °C until required for total RNA extraction and sRNA library construction.

### Triterpene saponin content analysis

Leaves, stems, and roots were collected from 1-, 2- and 3-year-old plants, and dried at 60 °C. Twenty samples for each year were used for triterpene saponin content analyses. Approximately 0.2 g of the dried material was ground in a 10 ml centrifuge tube, soaked in 2 ml methanol, and extracted twice with ultrasonication for 30 min, then cooled to room temperature. The residue was reconstituted with methanol for HPLC analysis according to Chinese Pharmacopoeia (2010 Edition). All analyses were performed in triplicate.

HPLC analysis was performed using an Agilent 1260 Infinity system (Agilent, Palo Alto, CA). Samples were separated on an Agilent ZORBAX SB-C18 column (250 × 4.6 mm, 5 μm particle size) using a H_2_O:CH_3_CN gradient from 19–36 % CH_3_CN at 1 ml/min over 60 min. The wavelength of measurement was set at 203 nm. Rg_1_ (standard 1), Rb_1_ (standard 2), and R_1_ (standard 3) were obtained from the National Institute for the Control of Pharmaceutical and Biological Products.

### sRNA library construction and deep sequencing

Total RNA was extracted from 1y, 2y, and 3y *P. notoginseng* roots using Trizol reagent (Invitrogen, Carlsbad, CA) according to the manufacturer’s protocol. At each stage, five *P. notoginseng* roots were randomly and separately collected to prepare three pools for constructing sRNA libraries. RNA quality and quantity were examined using an Agilent 2100 Bioanalyzer. Three sRNA libraries were constructed using previously described methods [44]. Briefly, 10–30 nt sRNAs were purified from a 15 % denaturing polyacrylamide gel and then ligated with 5′ and 3′ adapters. After being reverse-transcribed by Superscript II reverse transcriptase (Invitrogen), sRNAs were amplified by PCR, then deep sequenced using the Illumina Genome analyzer (Illumina) at the Beijing Genomics Institute (BGI, Shenzhen, China). All sRNA sequences from the 1y, 2y, and 3y *P. notoginseng* root libraries have been deposited in the NCBI Sequence Read Archive under accession numbers SRR1554324, SRR1555735, and SRR1556449, respectively.

### Bioinformatic analysis of sRNAs

After removal of adaptor sequences and filtering low-quantity reads from raw reads, the clean 18–30 nt sRNAs were mapped to GenBank (http://www.ncbi.nlm.nih.gov/) and Rfam (version 10.1) database (http://rfam.sanger.ac.uk), with a cut-off value of 0.01, and rRNA, tRNA, snRNA, and snoRNA were discarded from the sRNA reads. The conserved miRNAs were annotated by a nucleotide-nucleotide Basic Local Alignment Search Tool (BLASTn) search against miRBase 19.0, allowing no mismatches. To identify novel miRNAs, the MIREAP program (http://sourceforge.net/projects/mireap/) was used to obtain all candidate precursors with hairpin-like structures that were perfectly mapped by sequencing tags, with the following parameters applied. It includes minimal miRNA length (18), maximal miRNA length (25), minimal miRNA (reference) length (20), maximal miRNA (reference) length (23), maximal copy number of miRNAs on reference (20), maximal free energy (−18 kcal/mol), maximal space (300), minimal mature pair (16), maximal mature bulge (4), maximal duplex asymmetry (4), and flank sequence length (20).. The secondary structures of putative pre-miRNAs were then manually checked using Mfold [45]. The criteria chosen for stem-loop hairpins were described by Meyers et al. [46]. Target genes of miRNAs were predicted using the psRNA Target program (http://plantgrn.noble.org/psRNATarget) using a publically available *P. notoginseng* transcriptome (accession number SRP060270) derived from 1-, 2-, and 3-year-old roots. The threshold use for target prediction were using default parameters based on those suggested by Schwab et al. [47] and Allen et al. [48]. Target gene analysis was also performed using the transcriptome of Liu et al. [9], however, no additional genes putatively involved in biosynthesis of PNS was identified as targets.

COG, GO, and KEGG were used to investigate putative functions of target genes [20]. COG database is based on the evolution relation of protein system among bacteria, algae and eukaryotes. Protein sequence can be classified into one kind of COG parts and each kind of COG part is composed by homologues sequences which can be used to deduce the function of protein, with the e-value of 1e^−5^. GO is an international standardized classification system for gene function, which supplies a set of controlled vocabulary to comprehensively describe the property of genes and gene products. There are 3 ontologies in GO: molecular function, cellular component and biological process. The basic unit of GO is GO-term, each of which belongs to one type of ontology. The GO annotations were obtained based on the BlastX search against the *A. thaliana* database (from NCBI) with an e-value threshold of 1e^−6^. KEGG is the major public pathway-related database. KEGG pathway analysis identifies significantly enriched metabolic pathways or signal transduction pathways in target gene candidates comparing with the whole reference gene background. Genes with FDR (false discoverty rate) ≤0.05 are considered as significantly enriched in target gene candidates. The KEGG analysis could reveal the main pathways which the target gene candidates are involved in. The e-value threshold was sat at 1e^−10^.

To analyze differential miRNA expression, miRNA expression in each library was normalized to calculate the expression of transcripts per million. The fold-change and *P*-value were calculated from the normalized expression. Significantly differential expression was defined when |log_2_ratio| ≥ 1 and the adjusted *P*-value ≤ 0.01 [24–26].

### Quantitative real-time PCR analysis of miRNAs

qRT-PCR was used to validate the miRNAs identified using deep sequencing and to analyze their expression patterns. Total RNA of roots, stems, and leaves from 1y, 2y, and 3y *P. notoginseng* plants was extracted using Trizol reagent (Invitrogen) according to the manufacturer’s protocol. They were then reverse-transcribed into cDNA using the AMV First Strand cDNA Synthesis Kit (TaKaRa, Dalian, China) according to the manufacturer’s instructions. qRT-PCR was carried out on the ABI StepOnePlus RT-PCR systems (Applied Biosystems, Foster City, CA) using the amplification conditions recommended by TaKaRa and the ABI SYBR® Green PCR Master Mix (2×) (Applied Biosystems). Each sample was processed in triplicate, and 18S rRNA was used as an internal control. Primers are listed in Additional file 9: Table S7.

### Availability of supporting data

All sRNA sequences from the 1y, 2y, and 3y *P. notoginseng* root libraries generated by Illumina sequencing have been deposited in the Sequence Read Archive (SRA) data base (http://www.ncbi.nlm.nih.gov/sra) under accession numbers SRR1554324, SRR1555735, and SRR1556449, respectively. The mRNA sequences from the 1y, 2y, and 3y *P. notoginseng* root also have been deposited in the SRA database (http://www.ncbi.nlm.nih.gov/sra) under accession number SRP060270.

## Additional files

Additional file 1: Table S1.
*Panax notoginseng* saponin content from different tissues of different ages. (XLSX 9 kb) Additional file 2: Table S2. Conserved miRNAs in the three libraries of *Panax notoginseng*. (XLSX 24 kb) Additional file 3: Table S3. Novel miRNA candidates in *Panax notoginseng*. (XLSX 17 kb) Additional file 4: Figure S1. The secondary structures of new *Panax notoginseng* miRNA precursors. (PDF 44 kb) Additional file 5: Table S4. Statistics of target genes of novel miRNAs in *Panax notoginseng*. (XLSX 21 kb) Additional file 6: Table S5. Statistics of target genes pathways of novel miRNAs in *Panax notoginseng*. (XLSX 16 kb) Additional file 7: Table S6. The statistically significant differentially expressed known and novel miRNAs in *Panax notoginseng*. (XLSX 17 kb) Additional file 8: Figure S2. qRT-PCR validation of six differentially expressed miRNAs identified using Illumina small RNA deep sequencing. *A*. Fold-change of six miRNAs that were differentially expressed among 1-, 2- and 3- year old roots based on deep sequencing data. *B*. The relative expression abundance of the six miRNAs 1-, 2- and 3- year old roots by real-time quantitative RT-PCR. (TIFF 737 kb) Additional file 9: Table S7. Primers used for stem-loop qRT-PCR. (XLSX 9 kb)

## Abbreviations

AP2: APETALA2
BLASTn: Nucleotide-nucleutide Basic Local Alignment Search Tool
FDR: False discoverty rate
COG: Cluster of Orthologous Groups
CYP450: Cytochrome P450
DMAPP: Dimethylallyl diphosphate
DXP: 1-deoxy-D-xylulose-5-phosphate
DXS: 1-deoxy-D-xylulose-5-phosphate synthase
GO: Gene ontogloy
GPP: Geranyl diphosphate
GPS: Geranyl diphosphate synthase
HMGS: 3-hydroxy-3-methylglutaryl coenzyme A synthetase
HPLC: High-performance liquid chromatography
IPP: Isopentenyl diphosphate
KEGG: Kyoto Encyclopedia of Genes and Genomes
MEP: Methylerythritol phosphate pathway
miRNAS: MicroRNAs
MVA: Mevalonic acid pathway
MYB: Myeloblastosis related proteins
PNS: Panax notoginseng saponins
qRT-PCR: Quantitative reverse transcription PCR
rRNA: Ribosomal RNA
SPL: Squamosa promoter-binding protein-like
sRNA: Non-coding RNAs
snRNA: Small nuclear RNA
snoRNA: Small nucleolar RNA
tRNA: Transfer RNA
