# Supplementary material for: Identification of novel and conserved microRNAs in Panax notoginseng roots by high-throughput sequencing
Source: BMC Genomics. 2015 Oct 22;16:835. doi: 10.1186/s12864-015-2010-6 (PMC4618736; doi:10.1186/s12864-015-2010-6)
Supplement: Additional file 4: Figure S1. — The secondary structures of new Panax notoginseng miRNA precursors. (PDF 44 kb) [file 12864_2015_2010_MOESM4_ESM.pdf]

Figure S1 The secondary structures of new *Panax notoginseng* miRNA precursors.
(The mature miRNAs are in red)

novel_miR_1
GTTACATATGCCTATTTTGGGGGCGTCACAGTACGTACCATATGGGAGCAT
ATGAAGATGACAAGTCAATAAATGAGTACGGCCACCACATTGAAACGCAA
GCATGGATGATCCATTAGAATAGGACAAAATACATAGTACATACCATATGGG
AGCA
(((.(((((((((((((((((((((((.((((.(((..((..(((..((.(((..(..(((....))).....)..)))..))..)))...))..)))...)).))))))
.))).))))))))))....................))))))..))).
**********CCTATTTTGGGGGCGTCACAGT***************************************
****

novel_miR_2
CCTTGGCAGCTAGGAGTGAGCATTCGGTTATTAACCGAACCGAATAAGTA
TTTCGGTTATAACCGAAAACCGAACTTATACTGTTTTAGGT
(((.(((((.((.((((..(..(((((((((.(((((((.(......)...))))))))))))))))...)..)))).)))))))..))).
**********TAGGAGTGAGCATTCGGTTAT**
****

novel_miR_3
AACAGGTTTCTCTAAATACTGGAAGAGTTTGAGAATATTAAAGAAAACATTT
AGTATTCTCAAACTTTTCCAACATTTAGAGAAACCTGTC
.((((((((((((((((..(((((((((((((((((((((((.......)))))))))))))))))))))))..)))))))))))))))).
**********TCTAAATACTGGAAGAGTTTG**
****

novel_miR_4
GCTGTATAAACCTATTTTGGGGGCGTCACATCTCAGACCAGATCCGACCC
TAGATTGTTCTCGTCAATCTTGTATGCTTGCTACGACGTCGTCATGTCCTT
CGACCATTGCCAGCAGTAGTGATCTGTGTCTCCATCATCCACTTTGTCAAT
ATTTGAAAACATTCCGGCAGTGCAGCCAAAGATAGAGGCAGAGGATGTG
GCGCCGCTAAAGGAGGGGCATTACAGA
.(((((....(((.((((((.(((((((((((..(((((((((((.((...((((((.......))))))....((((.((......((((..........))))....))
.)))))).).)))))).))))..........(((((((..(((((...........(((......)))..)))))..)))))))))))))))))).))))))..)))....
))))).
**********CCTATTTTGGGGGCGTCACA***
****
****

novel_miR_5
GAGATGTACAGAAAGCTGTGGGTTTGGGGTGCTTGGGGAGGAAAGAAGT
GATAAATAAATAAATAAGAAACCATCCTTCCTAGTTCCTTCCCAAGATCCCC
CTAACCGAGGGTTTTTTTGTATTTTCCT
((((.((((((((((((.(.((((.((((..((((((((((((.((((.(((....................)))))))....))))))))))))..))))..)))).).
)).))))))))))))))..
**T

TCCCAAGATCCCCCTAACCGA*******************

novel_miR_6

TCTGCAGGGCATTTCTTTGGAAGTGGAGCTCCACACAGCTCACTGTTACC
GGCATATATCGAGTACTGATCATCAAGAGTGCGGAGCTACTGCCCTGTTG
GAATTCATTTGGTTGGAAAGATACCAGACAAGATCGGTGGCATGAACGCA
CTAGAATCAATTGACTTATCTAGAAACTAACTTTCTGGGGTATTCCTCCTAG
CATTTCAGGTATTATGACCTTTTTGAGTCACTTGAATGTTTCGTATAACAAT
TTGAGAGGGAAAATTCCACTGAGCAGACAACTCCAGAGCTTTAATGTTTC
AAAGCTTCATCGGCAATGCTGGACTTTGTGGAGCTCCACTTGCAAAGAAT
TGCAGTAGT

.((((...(((.(((((((.((((((((((((((((((....)))...(((((((...(((((((((..........)))))((((...((((.(.((.((((((..(((
((((.........))))))((((((.((((.........))))...)))..))).....((((((((.....))))))))...((((((..((((((.(((((.....(((
(.....).)))))))))))))................)))))).))))))).).))))....)))).(((((((........)))))))..))))..)))))))....)
)))))))))))))).))))))).))).)))).

****
****
****
***TGGAGCTCCAC
TTGCAAAGAA**********

novel_miR_7

GAACTTAATAAGTGAAGCTTGAGTATGACATGCTCAATCAACTCTTTAAGG
TTCGTGAGCATGTCATATTCAAGCTTCACTTGTTTAGTTTC

(((((.((((((((((((((((((((((((((((((...((((......))))..)))))))))))))))))))))))))))))).))))).

**********AGTGAAGCTTGAGTATGACAT**
****

novel_miR_8

ATTATATCTCAGGTTGCAGCGGCGAGGGAGCGGGGTTAAGGTCCTTGGG
TTTGATTTTGACCCAGATTAAAAAAAAATGGAAACCTCTTGCTTGGTGTAT
GTAAACCTCCAAAACCCC

..........(((((((((((.((((.(((..(((((....((((((((((.......)))))))............))))))))))).)))).))).))).)))))...
....

**********AGGTTGCAGCGGCGAGGGAGCGG**********************************
****

novel_miR_9

ATGGTTGTCTTTGTATTTTTCCACGGCTTTCTTGAACTGCATCGATCTGTC
TGTCAGTCAGTTTAATTAGGCCGATGCCATTGCCACAGTTCAATAAAGCTG
TGGGAAGATACAGAGAGGATCAA

.(((((.((((((((((((((((((((((((.(((((((((((((.((((.(((.....)))......)))).))))))).........))))))).))))))))))))
))))))))))).))))).

**********TTGTATTTTTCCACGGCTTTC***
****

novel_miR_10

GCTTCCTTGCTCCAATCCTTCTTGGTCCTTCCTTTGGATTTTGAATGACTG
ATACAAGGAAGAAGGATGGGAAGAGGAAG

.(((((((..(((.((((((((...(((((...((((.((......))))))...)))))))))))))).))).)))))))
***TGATACAAGGAAGAAGGATGG**
****

novel_miR_11
TTTGGCTTGACCTGCATTTGCGCCTGCACCTGACCGACCGTTTGCACACT
GCACGTACAGGTGCAGTGGCAATTGCAGGCTAAGCCAAT
.((((((((.((((((.((((..(((((((((..((......((((...))))))..)))))))))..)))).)))))).)))))))).
**AGGTGCAGTGGCAATTG
CAGG**********

novel_miR_12
TGCATCTAAAACTAAGATCAATGTAGTCGTATGTGAAACCAGAGTAATAGTT
TGATCAAATAAAGTCTCCTAGGCCTATTTTTAAGAATAGTGAATTACGAGGT
ACTATTTTCATTAATAGCATTTAACCAACCGGACACCACCTCATCACAATCA
TGTTGCAGGTTTCTTGCGAGCAAAGCGCTGCAATGATTTTGGTAGCTCAT
TTT
..........(((((((((.(((((.(((.(((((((((...(((((((.(((..........((((...))))((((((....))))))........(((((.(((((.
.....)))))).......((....)).....)))))....))).).)))))).))))))......)))..)))))))).))))))))))..........
**********ACTAAGATCAATGTAGTCGTA**
****
****

novel_miR_13
TTTCTCTTCAACTCCTACTCCCGCGGACGTTTCTGCTGCCGTCGCCAGCA
GAGACGTCGGCGGAAGTATGAGTGGAGGAGGGT
.((((((((.((((.((((.((((.((((((((((((((....).))))))))))))).)))).)))).)))).)))))))).
**********ACTCCTACTCCCGCGGACGTT***
****

novel_miR_14
TATGTGTCTTTTTGGATACACGCAACAAACAGGATTATGCAATTTATACAAC
CCTGTTTTTTGCGTGTATCCAAATGATACCTA
...(((((..((((((((((((((((.((((((((...................)))))))).))))))))))))))).))))))...
**********TTTGGATACACGCAACAAACA**
****

novel_miR_15
GAAACCATTGGAATGGGAGGAATTGGCAAGAAAAATAGCCAATTGAAGTA
GTTTATTTTTCTTGCTAAGCCTCCCACTACCTCTGCCTTCA
(((..((..(((.((((((((..((((((((((((((((((.(..........).)))))))))))))))))).)))))))).).))..))..))).
**********GAATGGGAGGAATTGGCAAGAA**
****

novel_miR_16
GGGAGTGAGCTGTTTGAAGATTACATTGGATAACTTGGAGGCGTCAATAC
CTGACAAATGCAGTGTTGCAGGCCAAGAGTGACACTGACAGTGATGGCC
AAGAGTATTGTACTAAGATCTTACTTAGGTCTCAGTACTAGGTTAATTTTAT
GGATAGTTTGGTTTTTTTTTTG
((((.((((((((((.((((((((..((((...(((..(((((((((.....))))...(((((((((...(((((..(.((.......)).)..)))))...))))))))

))(((((......))))))))))))).))))..)))))))..))))))))))).))))).......
****
***TAATTTTATGGATAGTTTGGT
****

novel_miR_17
CCACTCGGCGTCGAACTGCATGAAGGCGATGTCTAAAGCTTGCTCCAAA
GTATATATCCTCACATCTTTAGTGTGACGCCTCCAAA
......(((((((.((((.(((..((.(((((.....((((......))))..))))))).)))...)))).))))))).....
**********TCGAACTGCATGAAGGCGATG**
****

novel_miR_18
TGGATACTCGGTTTGTTTTTCGAACTCGTCTTCGGAAACATGGATCTCCAC
AGGTATGACGATCGGAGGATCAACTTCAACAGGGGGAACCC
.........((((.(((((((((..(((((..........(((....)))......))))))))))))).))))......(((....)))
**********GTTTGTTTTTCGAACTCGTCTT**
****

novel_miR_19
GTGGAAAATAGAGGAAAAGTCAGAACGGGTTTGGAGGGACCTGGACTCA
CTTTTGCTC
..........(((.((((((.((..(((((((....))))))).)).)))))).)))
**********GAGGAAAAGTCAGAACGGGT****************************

novel_miR_20
GCAAGTCCACAACTTTTTGTTGCATTTGCTACAAAGGTTGCATAGCGCTG
ACCCACAAAAAGGGTGTTAAATGAGAAAAATAGCGGACGCAACTTGTGCC
ATAGCAAATGCAACAAAAAGTTATGAACTTAC
..((((.((.((((((((((((((((((((((((.(((((((....(((((((((......))))...............)))))...))))))).))...)))))))))
))))))))))))).)).))))..
****
*************TAGCAAATGCAACAAAAAGTT**********

novel_miR_21
CCTTCGTTCTTCAGTCTCAGTAAGTAGCCGCGGCTATAAATTTCATAGCAG
CGGCTACTTACTGAAGTAAGAATTGAGAGA
((((.((((((.(.(.(((((((((((((((.(((((.......))))).))))))))))))))).))))))).))).).
**********TCAGTCTCAGTAAGTAGCCGC**
****

novel_miR_22
TGGAGGGGATCAAGGGCTTCGATTTCTCTCTCAACGATTTTGTAGCAATC
GATTTCAAGGAACTCGCTCGATTTTAGCAGCTTCGATTTTACAGCAATGTG
TTTTCTGGAGGGGAAACGAGAGCTAAGTCGTTCT
.((((.((......(((((((.(((((((((.(((((((((((((.((((((.......(((.((....)).)))......))))))))))))).))).)))))....)))
))))))).))).)))))...)).))))
****
****************TCTGGAGGGGAAACGAGAGCT**********

novel_miR_23
GTAGCTCAGTTTGCCTAAAGCGAGATTCTCAGTTTGCAGAACCTAGAATC
GCGCCCAAATCGTCTGCCCTGAGAATCGCGATTTAGGCAAGGCTGGTTG
A
.((((.(((((((((((((.((.(((((((((...(((((....................))))).))))))))).)).))))))))).)))))))).
**TGAGAATCGCG
ATTTAGGCAA**********
novel_miR_24
ATGAGAAATCGGGAGCATTTGGGTTGGGAGCGACGGGAAAGGCGATTGA
TAATAGGGAGCATTTGCCTTTCCTATTCCTCCCATACCAATTGTTTCCGATC
GTTCCTCC
..(((..((((((((((.((((..((((((.((.(((((((((((.((...........)).)))))))))))..))))))))..)))).))))))))))..)))....
***TTTCCTATTCCTC
CCATACCAA********************
novel_miR_25
CAAGTAACAGTTTAAGTTTGGGGGTATTTGATAAGTAGTTTTTATACTTACT
TATCCAGTATCTATTACTTGAATTAGTTGCTTT
.(((((((((((((((....(((((((.((((((((((......)).)))))))).)))))))...))))))))).)))))))).
**********TTTAAGTTTGGGGGTATTTGA**
****
novel_miR_26
ATGTAGACAGAGGCGGTGATGATGAAAATTATACCATCAAACTTTGAAAGC
TTAAGAGCTTGTTGATGCCAACAATAATCATTACAACCTGAGCCTCCTAGT
TTATGTT
((((((((.((((((((..((((((..(((....((((((.(.....(((((....))))))))))))).....)))..))))))..)))...)))))...)))))))).
**********AGGCGGTGATGATGAAAATTA**
****
novel_miR_27
ACTGTGACTGAGTGAATGAAGCGGGAGATAGTTTTCTATCCCTCCTCCTC
TGTGCTTGGACTGAAGGGAGCTCCCTTATTCTGTTGCCCGCTTTCATTTT
CATCTT
...((((..(((((((...((((((.(((((..........(((((..((.((......)).)).)))))..........))))).))))))))))))))))...
**********AGTGAATGAAGCGGGAGATAG**
****
novel_miR_28
GTTTCCAGAGCCCGGCATGTTCATCATTGTATTAATTCGAATTGCTTTTATT
TGTTGATTTATAATTTTAAAGAATTAGTTGCTGTGTAATTTGATGAAAATTTG
GAGAAGATGAATATTTTGATAAAAGGAAA
.(((((......(((.(((((((((.((.(...((((((((((((......(((.(..((((......))))..).)))........)))))))))).....)))...).)))
))))))).)))......)))))
****
*************TTTGGAGAAGATGAATATTTTGA**********
novel_miR_29
GTTCCCTTGTCCAACCAGATTTCCTGAAAAAATCAAGTCTTTGAGCTGATA

TTTCCAGGCCTTGTGAATCCAATTTGATTCTAGGTACTTTCTCTATGCTCTG
GATCTTACATTAGGGGCTGGATATAAGAAGAGATTTGAGGTTAGAATTAAG
GTTGGGTATGAGAGATAAGAGATTTAGGAGAGTTGAGAGAGATCGAGTTG
TTTGGGGGAA
.((((((((..((((..((((((((.((....((((((((((...((..(((.(((((.(((((..(((((((.((..((((..(((.....((((.(((((.(((...
..))).))))))))))))).)))))..)).))).))))).....)))))))))).)))..))...)))))))).))....)).)).))))))..)))))..))))))))
****
****
***************AGAGATCGAGTTGTTTGGGGG**

novel_miR_30
GGCGGAGCCAGTCTTGGCATTGCGCTGGCATTGCGCGGCCGGCATTGC
CGGCGGAGGCCTTCTAGCTGCGAA
..((.(((.((....(((.((.((((((((.(((.......))).)))))))).)))))..)).))).))..
GCATTGCCGGCGGAGGCCTTC******
*

novel_miR_31
TCTGTTTGTGACTTTTAATTTGGGGGGAAGGGAAGGGATGGGAAGGGAA
CATATTTCTTTCCAAAAATTTCGGAGAAAGAACTTCCCCATCCCATCCCTT
CCCCTTCCATCCGAAACAAAACTC
....(((((.........((((((.((((((((((((((((((.(((((....((((..(((.........)))))))....)))))..)))))))))))).))))))).)
))))))))))....
****
****CATCCCTTCCCCTTCCATCCGA**********

novel_miR_32
GTATGGTTGAAGAAGTAGAACAAGAAGATGAAGAGGGATATGATGCTTCA
GCCTTCAGTCATGAAGAATGCAAAAATTCAGCTGCCGCAGCCGCCGCCC
TCATCTTCTTCTTCTACTTGCTCCACCAAAA
...((((.((..((((((((.((((((((((...(((......(((..(((((((((....)))))).............))))..)))........)))))))))))).)
))))))))..)).))))...
**********AGAAGTAGAACAAGAAGATGA***
****

novel_miR_33
TTGAGGACTGCAGTTTTCGCCATCAAAGTCATGACCACAATTTAACCTTAC
TGCAACGCCCGGCCATATTTCATTGTGATGCTTGTGACCGTGTAGCAGAG
GACTTGTCTTATC
...(((((...(((((((((...((..((((((((((((((.........(((.......))).........))))))..).))))))))..))..)).))))))))).)))))
...
**TGT
GACCGTGTAGCAGAGGAC**********

novel_miR_34
TATGCTGTTTTTTTAGTAAGAAAGTGGAGCAACATCTTCTGAGTGCAAAAA
CTTGATTTTCATTCTAAGAAGATGCGTGCTCCACTTTCTTAATAAAAAACAG
CATAT
((((((((((((...((((((((((((((.(((((((((((((.((((......)))))))))..))))))))..))))))))))))))..))))))))))))

).
**********TTTTAGTAAGAAAGTGGAGCAA**
****

novel_miR_35

ATTAATTTCTCCATCACTTTCTTTAATTTTTAAGATATAAGTAAATAGAAAAAT
ATAAGGTGAAGAAGGACTACGTACTAGTACTAGTACTACTGTAATGGAACT
CCCGTTTAGTAGAAGCTTAAGGAGCAGAAGGTCTCATGACAAGAGAAGG
GATGGATACATCCGAGTTGATGCAACCGCAGATGATGATCATGAGGAGGG
GAAGACGATGAAGACGAAGATGAAGATGAAGATGAAGATGATTAGGGCG
CC
.((((((((.((((....((((((.(((((....(((.((((..(((.....(((..(((........)))..)))))).)))).)))((((((.(((((.....))))
)))))))..((((...))))......((((.......))))..(((((....)))))..(((..(.(..((.(((((....))).)).))..).)..)))........)))
))))))))....)))).))).))))))......
****
****
GATGAAGATGAAGATGAAGATGA
****

novel_miR_36

GATAGTTTTGGGAAACTAAAGGTAGTAGGTGGTTTGGAAAGACTAATAAGA
AGCTCTCTTAGCTTCTTCATATTCAATTATATTTATTTGGTTGGGTTTGAAGT
AGTACACGTCTTTCCTAGACCGCCCATTCCTCTGGTTTCCTGAGATTACTC
C
..(((((((((((((((.(((.(((.(((((((((((((((...(((((.....)))))(((.(((((.(((((((((........))))))))).))))).))).
...))))))))).))))))))).)))))).))))))))))))))))....
****
**************************TTTCCTAGACCGCCCATTCCTC********************

novel_miR_37

CACCTACAAGTGAGAGAGAACCATGTGAAGATATAAAAAACTCGTGTGGA
AATAGTGTTCCTCTAAAAATAAAGGGCAAGTGGAAAATTTAATATCAGAGCT
CTCTCGGCCATCCGAGGAGAAAAGCCTTCCCTCTTTCTAGTCTGAATTGA
TAGACTAACGCTCCACAATAACTTCATCAAAATTCCAGTCTCTCACAAGGT
CTCTTCTAACAGCACGGCTGCGAACTAGCCTTGGGGGAC
.((((....(((((((((((...(((((((.(((..........((((((....(((((..((((..(((...((((...((((((.............(((.(((((...
.))))))))..............))))))))))..)))..))))..)))))))))))))).))))).))...)))...)))))))).))))).((((((((......((((
(.....)))))))))))))).
**********TGAGAGAGAACCATGTGAAGATA**************************************
****
****

novel_miR_38

CAAACTTTTGCATCGGGTCTTGCCGTGGTCTCTCAGCATCGATGACAGTT
TCTCCTGTCCTTTGGTTTCGTGCTTGACGTCTTGGCGGCATTATCTGGTTA
AATAAAAC
......((((.((((((((..((((((.(...(((((((((((.(((((......)))))..)))).....)))).))).)...).))))))..)))))))))))......
***ACGTCTT

GGCGGCATTATCTGGT**********

novel_miR_39

TGAAGGGACATTTGAAGAAGGGAACAAATGGGAGTTGAATATATAATTTTT
ATAAAGAGCATTATACTAAATTTTACAAGTTGAATGGACTGTCGTATGTCTT
CCGAGTTTTCTTGTTTGAAACCATTCCGACAC

.(((.((...((((((.((((((((...((((((....((((((.((........((..((((..(((..........)))..))))..)))).))))))))))))).)))
)))))).))))))).)).)))......

**********TTTGAAGAAGGGAACAAATGGG***************************************
****

novel_miR_40

GGAAGATATCAATATGTTGGTAGCTCGTTTATCGCAGGATGGACCCGACG
GATCCGTTGTGGCTATTTATGGGATGGGCGGTCTGGGAAAGACCACCCTT
GCTAGAAAGATATACAATCACCCGACGACTAAGCGACATTTTGCTTGTCTA
GC

...(((((.(((.((((((.(((.(((((.(((.((.(((((.(((((((....))))).)))))))..)).)))(((.(((((.....))))).)))..........(
((.....)))....))))))))..)))))).)))..)))))...

****

**********************************CGACGACTAAGCGACATTTTG**********

novel_miR_41

GCGGCTGCCCCTGCCTGCCGTAGCCGTTCCCTTTTTGCCCTAATCGCATT
AATAGCGGCCTGATCAAGAATCACTGACCCGGATGACTCGTTCCCTTGCT
TTAACACTTTTTCTTTCCTGAACGAAGTAGTATCATTTGTGTCATCCTCATC
ATCATCAGAATCACCTGTGTTGGTGCTATTATTGAGGGGTTTTATTAGGCC
CTTCAGAATTATTACAGGCTCCGAGGGAGGAGGGTTACGGGAGCTAACA
GCCAGGGTTTTGGTGTTCTTTTGGAGCTCGAGGAGAGCTTCCTTGGTGT
AAGTTCCCGCTTGGGGTTGGACATTGGAAGAGAATGAAGACGTGGGCGG
CGGAAGGCGG

.((.((.((.((((((((.((...(((((.(((((.(((((((((((.......)))).)..((((.....)))).((((((((((((.((......((((.....(((((
(((......))).)))))))))).....)).))))))).(((((.((.(((......))))).)))))......((((((((((((....)))))))))))...........
((((...))))..))))))((((((((.(((.((((((.......((((((((.(....).))))))))..))))))))).)))))))).)))))))........))))
).)))))...)))))))))).)).)).)).

****

****

****

***AGAGAATGAAGAC
GTGGGCGG**********

novel_miR_42

ACAAGAGAGACTTGTTGTTCTGTTGAGGCAATCTTACAGTTCTTGAAGGA
GGAGGGGTATGATGTGACAATGTCAAATGATGCGGGTCGTTATGTTTGCA
ATTATGTATATTATCACTCTCTCCGGTTTGCAGAACAGAAAGGTCACAAATC
TCTTTT

..((((((((((.(((((((((..((((..((((.((.....)).))))((((((((((((((((....((.(((((...(((...)))...)))))))......)))
)))))).)).)))))).)))))))))))))..)))))).....))))))..

****

TTGCAGAACAGAAAGGTCACA********

novel_miR_43

CAACGAATCTTTGGGTCAGTATGTGAAACGGTTCCGAGAGGCCGTTGGT
CAGGTATCTGATTTGAACGATCTCCAA

......((((((((((((((((.((((((((((......)))))))..))).))).)))))))))).)))......

**********TTGGGTCAGTATGTGAAACGGTT***

novel_miR_44

CTTGACCAAGTCCATCGCAACTTATCTTTCCTCTTCAACATTAGATGCAAA
ATCTCCGCCTCCTTCTGGGACACAATCTTCTGTTGGAACAGAGGAAACAG
TGCAATTCATGATGGTTAGC

.(((((((.((..((.(((.((....((((((((((((((..((((......((.(((........)))))....))))..))))))...)))))))).))))).))..)
)..))))))).

****
AGAGGAAACAGTGCAATTCAT********

novel_miR_45

GTAACACTAATGTTGTGTGTGGTTGGTGGGAATTAAATGGAATGGACTGG
GAATGGAATGGCTTAAAACATGTTTGGTTGATCAATTGGAATGAGCATTCC
ATCACCTTCTCTCGCTACCTTTCTCCCATCGATCGAAGAAGAAAATTAGCA
CCAA

......(((((.((.(...(((((((((((......(((...(((..((..((((((((.((((...((...(((......))).))...))))))))))))..)).)))
....)))......)))))))))))....).)).)))))......

**********TGTTGTGTGTGGTTGGTGGGAA**
****

novel_miR_46

TTTGTTGTATTAGTATAGATAGAGAGTTGCATTTCGATAATCTTTATTATACA
AAGAATATATAATTGCAGCTCTCTAACTATACAAATAAACTCA

...(((.((((.((((((.((((((((((((.((..(((.((((((.......)))))).)))..)).)))))))))))).)))))).)))))))...

**********TAGTATAGATAGAGAGTTGCA**
****

novel_miR_47

TGAATCACCCTTTCTCCCTGTTTGTTCTCAGATTCTAGTTTTCAATCTAGAA
CAAAAGAATATTGTTGGATTATTTAAGGAGAGAGAGAAAGATTGATAGATAC
AGAAATAAAAGATAGAAGGGGAAGGGGAAGGGGAAGGGGTTTTGAG

......((((((((.((((.(((.(((((...((((((........))))))........((((..(.((((.(((...........))).)))).)..))))..........
.........))))).))).))))))))))))......

****
*******************************AAGGGGAAGGGGAAGGGGAAG**********

novel_miR_48

AAGTTGAATGAAGTTGAAATTGAGCAACTTGACGCCGGCAAGACTCGTG
CTAAAAGGCTCATTATATAAGATTTAAGCAAAATGGTCAAATTAAAGCTAAAT
GCAAAATATTGTGGAGCTATTTTAAGTGCTGATCCAGAAAATAATGGGACT
AGTGGTCTAAATGCTCACAATAGGAGATGTAAAATTTACCCCCCAAATGTG
GAGTTAAGAAATCAAGCAATCTTAAACT

.((((...(((.((((...((((....((((((.(((.((.((((..((((.(((...............))).))))....))))........((.....))....(((((
((.(((.((((..(.(((((.((((........))))..))))).)..)))))))))))))(((((.....)))).)).......))))).))))))...)))).)))
).)))))))
****
****
***********************ATGTGGAGTTAAGAAATCAAGCA**********

novel_miR_49

ATTGGTAAAGGTATATCGCCAGATGTTTACAGCTATAGTTCATTAATTCAGG
GCCTGTGCAACTTTGGTCGATGGACAGAGACTGA
.(((((....((.((((((((((.((((((((.....((((.........))))))))).)))))))).))))).))....)))))
**TGTGCAACTTTGGTCGATG
GA**********

novel_miR_50

ACCAAAGTCTGATATTTTCACTTCCATTTTCGAGTTTACCAGGAGATTAGC
GGGTTTAATATCTCTGTGAATGATTTTGTGGGAGTGGAGATAGCTAAGG
.((..(((....((((((((((((((......((((((..((((((.............))))))))))))......)))))))))))))))))..))
**TGTGGG
AGTGGAGATAGCTAA**

novel_miR_51

TGTTGCTTAAAGTAGACATGCCTTTGTTGAACCTAAAATGTCAATCACTCTA
AGGACTAACAGAAGGAATGTTACATCATCATTAAACCTCAATCCCTTATTAT
AATTTTGTAATTAAATTTGAAGACATTTTTCAACGAAATGAAAGCAAATTAG
TGATATGGTAAATAGAGCAAGT
..((((((...((.(((((.(((((((((..(((....((.....)).....)))..)))))).))).)))))))...................((.((((((...(((((
..(((...(((((((...))))))).....)))..)))))...)))))).))......))))))..
**********AGTAGACATGCCTTTGTTGAA***
****
****

novel_miR_52

GCTTATCACCTTTTATGGAAGTTGAATGACAAAGTATTACTTATTTATCCAAT
TTTTGAAGGAACAAAAGTTGTTCAATACTTAAAATCTCTTTCAGAGACTTTT
CTGTATTGAGTGTGGCAAAGA
(((...(((((((((..(((((((.((((..((((...))))..)))).))))))))))))).((((...))))(((((((..(((((((((...))))).))))..)
))))))))).)))).....
**********TTTTATGGAAGTTGAATGACAA***
****
