# Supplementary figures and images for: Identification of novel and conserved microRNAs in Panax notoginseng roots by high-throughput sequencing
Source: BMC Genomics. 2015 Oct 22;16:835. doi: 10.1186/s12864-015-2010-6 (PMC4618736; doi:10.1186/s12864-015-2010-6)

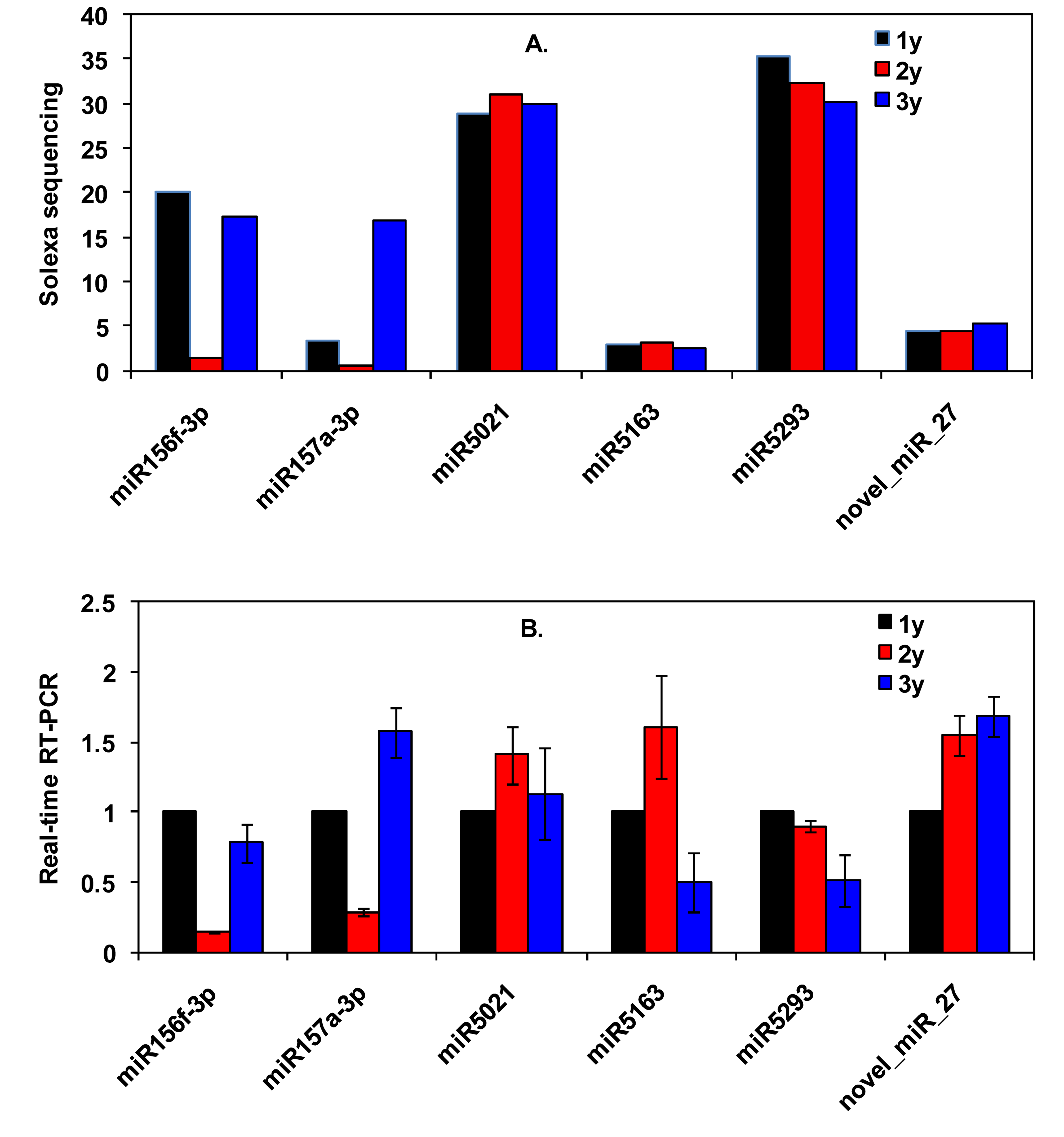

Supplement: Additional file 8: Figure S2. — qRT-PCR validation of six differentially expressed miRNAs identified using Illumina small RNA deep sequencing. A. Fold-change of six miRNAs that were differentially expressed among 1-, 2- and 3- year old roots based on deep sequencing data. B. The relative expression abundance of the six miRNAs 1-, 2- and 3- year old roots by real-time quantitative RT-PCR. (TIFF 737 kb) [file 12864_2015_2010_MOESM8_ESM.tif]
